# Multiple intertwined crises facing humanity necessitate a European Environmental Research Organization

**DOI:** 10.1111/1751-7915.14054

**Published:** 2022-03-22

**Authors:** Kenneth Timmis, Willy Verstraete

**Affiliations:** ^1^ Institute of Microbiology Technical University Braunschweig Germany; ^2^ Center for Microbial Ecology and Technology (CMET) Ghent University Ghent Belgium

## Abstract

The planet is experiencing all manner of environmental crises, and crises that have their origins in the environment, including global warming, pollution of the air, soil, marine systems and freshwater, loss of habitats and species extinctions, transmission of deadly animal infections to humans, the spread of antimicrobial resistance, to name just a few. Planetary boundaries are being successively breached. Devising and implementing optimal solution and mitigation strategies urgently requires the best possible scientific brains to be harnessed and focused on environmental crises. It is imperative to establish authoritative leadership and the intellectual and organisational framework for this: a European Environmental Research Organisation, modelled on the European Molecular Biology Organisation (EMBO) and Laboratory (EMBL), whose mission is to carry out pioneering research on environmental crisis‐relevant topics, communicate its findings and recommendations to governments, their agencies, the general public, business and other stakeholders, and create outstanding research leaders to populate the best institutions worldwide – a global network of top scientists working together to understand the causes and nature of crises and to devise effective solutions.

In 1987 – 35 years ago – a group of leading European environmental microbiologists came together to create the European Environmental Research Organisation, the EERO. The main driver for this was the rapid deterioration of the environment due to rampant pollution, and the urgent need to understand environment functioning and parameters impacting its health, in order to develop ways and means to protect and repair it, and provide authoritative advice on its future care. The guiding light for the creation of the EERO was the inspirational model of the European Molecular Biology Organisation, the EMBO.

The philosophy of the EMBO was simple (https://www.embo.org/about‐embo/mission/): first, to award highly competitive postdoctoral fellowships to the best young molecular biologists to work in the best research laboratories, with the aim of creating the next generation of world leaders in the field, and second, to establish the European Molecular Biology Laboratory, the EMBL, in Heidelberg, to provide the best facilities and intellectual environment in the world for pushing forwards the frontiers of molecular biology, and to act as a voice and beacon for the field. Although a European organization, science is international, and these molecular biology stars went to all corners of the world and provided outstanding leadership globally. Because molecular biology underlies all biological research, including medicine and agriculture, the EMBO and EMBL pioneered experimental methods and approaches that impacted a wide range of disciplines. The influence of the EMBO has been and continues to be enormous.

The EERO had exactly the same goals as the EMBO/EMBL, but in the area of environmental research (https://pubs.acs.org/doi/pdf/10.1021/es00079a603), and aimed to repeat its international success. Since the environment impacts so many fields, environmental research has a similar broad generic value, relevance and impact as that of molecular biology.

However, although the EERO secured start‐up funding for the first five years of its existence, in particular generous contributions from a RABO‐Bank‐led consortium of ministries, research agencies and institutes in the Netherlands, where it established its headquarters, the Volkswagen Stiftung, the Swiss Department of Foreign Affairs and the Spanish Ministry of Education and Science, it did not succeed to secure sustained, European governmental‐level funding of the type that has supported and sustained the EMBO. The standard response to requests for such support was ‘we would never again make such a long‐term commitment’. Eventually, the EERO was absorbed by the European Science Foundation and soon thereafter ceased to exist.

Of course, the environment has never had the power to focus minds like cancer, heart disease, etc., which are very personal and immediate. For urban dwellers especially, the environment is often quite abstract, not on the radar screen.

Until now.

Now, we have extreme weather patterns, wildfires raging, melting glaciers, flooding and landslides, melting permafrost and microbial release of the locked‐up carbon, carbon locked up for centuries and millennia in peatlands and ancient forests now being released by wildfires and deliberate burning (not to mention gas clathrates, which are just waiting for their release), all of which are amplifying global warming driven by the release of greenhouse gases from human use of fossil fuel. All of which lead to significant loss of life, property and progress in economic development. The environment is finally becoming more personal and more immediate for many. In addition, we now have COVID‐19, which is even more personal and immediate to dense populations – urban dwellers – caused by a virus that originated in bats and is spread by aerosols, both environmental processes.

Today, the planet is experiencing all manner of environmental crises and crises that have their origins in the environment, including

- global warming, driven by greenhouse gas emissions, a significant fraction of which has an environmental origin and a significant fraction of which is captured by environmental microbes
- pollution of the air, soil, marine systems and freshwater (including drinking water supplies), by toxic xenobiotics, heavy metals, powerful medicaments and pesticides, plastics/microplastics, etc., some of which are powerful endocrine disruptors that *inter alia* reduce fertility in animals, including humans (e.g. see Ghassabian *et al*., 2022)
- pollution of aquatic systems by agricultural fertilizers that cause eutrophication, the overgrowth of water bodies by photosynthetic microbes that are often toxin‐producers and that result in oxygen minimum zones and the associated loss of wildlife
- loss of habitats and species extinctions
- increasing transmission of animal infections to humans – zoonoses – some of which are highly dangerous, such as HIV, MERS, Nipah virus, Lyme disease and Zika virus, some of which have the potential to become catastrophic pandemics, like SARS‐CoV‐2
- the spread of antimicrobial resistance among environmental microbes and subsequent transfer of resistance to human pathogens, making them difficult or impossible to treat (https://amr‐review.org/sites/default/files/160525_Final%20paper_with%20cover.pdf)
- over‐exploitation of natural resources
- successive breaching of planetary boundaries (Steffen *et al*., 2015)

And the list goes on!

Imagine that an authoritative research organization with the stature and influence of the EMBO had been initiating, supporting and carrying out pioneering research on these and other environmental topics, and communicating their findings and recommendations to governments, their agencies, the general public, business and other stakeholders. A centre of excellence selecting and nurturing environmental research leaders to populate the best institutions worldwide: a global network of top scientists working together to understand the causes and nature of crises. It really could have played a major role in identifying emerging environmental problems and potential solutions, and thus been a considerable force in preventing and mitigating the crises we now face.

If those governments supporting the EMBO had drawn the obvious conclusion from their experience with it, namely that the EMBO had been an unprecedented success, not only scientifically, but also in terms of benefit to society, they might have done the same for the environment.

It makes sense to build on success, right?

Wrong: governments worldwide hate to make long‐term commitments, especially international commitments (witness the agony of reaching adequate commitments in COP26).

But, like it or not, the world’s governments hold collective responsibility for planetary stewardship and for the survival of the surface biosphere of the planet. In addition, success (and, just to be perfectly clear: success here means the ability of the planet to allow survival of our children and theirs – time is not on our side, see Timmis and Hallsworth, 2022) can only work through long‐term strategic planning and the commitments needed to implement the planning. The creation and nurture of organizations and agencies charged with research on those issues central to survival of the planetary biosphere – understanding underlying mechanisms, predicting trends, developing and testing hypotheses, trying new diagnostic tests and tools, prophylaxes and remedies, and the implementation of successful strategies to prevent, mitigate and repair damage – is imperative and pivotal to survival of humanity.

We desperately need to break down barriers, silos and compartmentalization in society that prevents collective effort and decisioning: Wildfires may seem like the business of forest agencies, COVID‐19 may seem like the business of health systems, polluted drinking water may seem like the business of local authorities, the submergence of low‐lying islands may seem like someone else’s business, but in fact all these, and many other issues, are also the business of the environment and those charged with its care, and a host of other stakeholders. So, it really is time to have environment‐centric multilateral investigation and management of a whole swath of problems.

Just one example that is on everyone’s mind right now: pandemics. The focus is on preventing disease and especially transmission, the main strategy of which is vaccination, so the main players are health professionals (vaccine administers), governments (vaccine gatekeepers and payers) and pharma (vaccine producers). Essentially, all the focus is on the disease. But, SARS‐CoV‐2 came from the environment: it is a zoonotic infection whose likely origin is bats. How it transferred from bats to humans is not presently known, but this information is key. An understanding of how pathogens circulate in wildlife, how they evolve to infect humans, how they transmit to humans, and how transmission can be reduced, are all pivotal to prevention and control of future pandemics. This is fundamental ecology – environmental science – not the realm of pharma, clinical medicine or government.

What will happen to SARS‐CoV‐2? No‐one knows, but it is an environmental issue: It is circulating in other forms of wildlife, like the white‐tailed deer (Chandler *et al*., 2021; Kuchipudi *et al*., 2021), and has also been detected in domesticated animals, some of which we infect ourselves, so elimination from humans through vaccination, even if this were possible, will not rid us of COVID‐19. How to suppress SARS‐CoV‐2 in wildlife? Culling is not possible, either practically or environmentally. Vaccination is theoretically an option and has been successful with another problematic zoonotic infection, rabies. But, rabies is spread primarily by foxes and vaccine‐loaded meat baits are eaten with relish by foxes. Deer are mostly vegetarian, so this will not work. But, who knows, perhaps, it will be possible to engineer plants eaten by deer to carry the genomes of live vaccines that are activated during digestion to produce infectious particles that engage the deer immune system? And to seed the plants at sufficient density in deer grazing areas. But, this is an environmental issue.

In addition, the next zoonotic pandemic is brewing in the environment. Whether it will be a porcine deltacoronavirus (Lednicky *et al*., 2021) this time or not remains to be seen, but the pathogens are actively exploring transmission and host range space, and testing our defences.

There are numerous other examples of environmental issues that impact our health and that demand environmental solutions, such as: every summer, during warm spells, many municipal and industrial wastewater treatment plants become infested with *Legionella pneumophila* (Caicedo *et al*., 2019), reaching loads of up to 10^8^/L in the wastewater and 10^9^/ m^3^ in the air above. *Legionella* can be detected up to 10 km downwind of such plants (https://www.watertechonline.com/wastewater/article/14069575/legionella‐growth‐and‐health‐risks‐from‐wastewater‐plants‐for‐workers‐and‐downwind‐communities). At such times, the whole aerobic wastewater treatment industry may become a source of *Legionella* emissions and, perhaps in some cases, infections (Kusnetsov *et al*., 2010; Vermeulen *et al*., 2021; https://www.rivm.nl/en/news/control‐measures‐against‐spread‐of‐legionella‐from‐wastewater‐treatment‐plants). The need to address this problem is urgent but is hardly attracting attention.

– the cultivation of corn/maize for fodder and for grain has become widespread in Europe. Hot summers increasingly result in crop infestation with aflatoxin‐producing *Aspergillus* species, thereby creating mycotoxin‐contaminated animal feed (Kumar *et al*., 2017, 2021). And mycotoxin‐contaminated crops and food are not limited to Europe; they are a serious problem worldwide (https://www.who.int/news‐room/fact‐sheets/detail/mycotoxins).

It is obvious that we need an EERO, to show leadership in environmental research, to demolish silos and compartmentalization that represent barriers to communication and synergies, and to provide answers and guidance relating to the survival of the biosphere. COP26 did not have much to say about research. With all the catastrophes humanity is facing, it is time to focus on a key strategic element of the problem and solution, namely environmental research, and to create the institution needed for this: the EERO. ‘We would never again make such a long‐term commitment’ hardly seems appropriate to the current problems faced by humanity and the planet. We need the EERO now more than ever, also to provide leadership in future COP meetings.

**Dedication**

This is dedicated to that wonderful band of seers who created the EERO 35 years ago with such foresight, energy and determination, especially those who are no longer with us: Bernard Witholt, Ivano Bertini and Bernard Dixon.
